# Rational Engineering of Recombinant Picornavirus Capsids to Produce Safe, Protective Vaccine Antigen

**DOI:** 10.1371/journal.ppat.1003255

**Published:** 2013-03-27

**Authors:** Claudine Porta, Abhay Kotecha, Alison Burman, Terry Jackson, Jingshan Ren, Silvia Loureiro, Ian M. Jones, Elizabeth E. Fry, David I. Stuart, Bryan Charleston

**Affiliations:** 1 The Pirbright Insitute, Pirbright, Woking, United Kingdom; 2 Division of Structural Biology, The Henry Wellcome Building for Genomic Medicine, Headington, University of Oxford, Oxford, United Kingdom; 3 Animal and Microbial Sciences, University of Reading, Whiteknights, Reading, United Kingdom; 4 Diamond Light Source, Harwell Science and Innovation Campus, Didcot, United Kingdom; Institut Pasteur, France

## Abstract

Foot-and-mouth disease remains a major plague of livestock and outbreaks are often economically catastrophic. Current inactivated virus vaccines require expensive high containment facilities for their production and maintenance of a cold-chain for their activity. We have addressed both of these major drawbacks. Firstly we have developed methods to efficiently express recombinant empty capsids. Expression constructs aimed at lowering the levels and activity of the viral protease required for the cleavage of the capsid protein precursor were used; this enabled the synthesis of empty A-serotype capsids in eukaryotic cells at levels potentially attractive to industry using both vaccinia virus and baculovirus driven expression. Secondly we have enhanced capsid stability by incorporating a rationally designed mutation, and shown by X-ray crystallography that stabilised and wild-type empty capsids have essentially the same structure as intact virus. Cattle vaccinated with recombinant capsids showed sustained virus neutralisation titres and protection from challenge 34 weeks after immunization. This approach to vaccine antigen production has several potential advantages over current technologies by reducing production costs, eliminating the risk of infectivity and enhancing the temperature stability of the product. Similar strategies that will optimize host cell viability during expression of a foreign toxic gene and/or improve capsid stability could allow the production of safe vaccines for other pathogenic picornaviruses of humans and animals.

## Author Summary

Picornaviruses are small RNA viruses, responsible for important human and animal diseases for example polio, some forms of the common cold and foot-and-mouth disease. Safe and effective picornavirus vaccines could in principle be produced from recombinant virus-like particles, which lack the viral genome and so cannot propagate. However the synthesis of stable forms of such particles at scale has proved very difficult. Two key problems have been that a protease required for the proper processing of the polyprotein precursor is toxic for host cells and the empty recombinant particles tend to be physically unstable in comparison to virus particles containing nucleic acid. This is particularly true in the case of Foot-and-Mouth Disease Virus (FMDV). Here we report the production and evaluation of a novel vaccine against FMDV that addresses both of these shortcomings. Importantly, the strategies we have devised to produce improved FMDV vaccines can be directly applied to viruses pathogenic for humans.

## Introduction

Foot-and-mouth disease (FMD) is a highly contagious viral disease of cloven-hoofed animals including cattle, sheep and pigs. Infection spreads rapidly through susceptible populations and can give rise to large scale epidemics, causing debilitation, pain and loss of productivity. Outbreaks of FMD such as that in the UK in 2001, which resulted in the slaughter of over 6 million animals and cost in excess of £8 billion, highlight the need for vaccines that support a ‘vaccinate to live’ policy. Vaccination is currently reliant on the use of inactivated virus produced in large bioreactors in high containment facilities. This is unsatisfactory on several grounds: the set-up and running costs are very high, limiting global production capacity, storage and supply are constrained by the poor vaccine stability at ambient temperatures, and it can be difficult to distinguish vaccinated from infected animals. Thus, more options for FMD vaccine production are urgently required.

FMDV is a non-enveloped single-stranded RNA virus belonging to the family *Picornaviridae*. The capsid consists of 60 copies each of four structural proteins (VP1–VP4). During assembly a 95 kDa polyprotein (P1) is cleaved by the viral 3C protease to yield VP0 (36 kDa), VP1 (32 kDa) and VP3 (27 kDa) which self-assemble to form the capsid. Auto-catalytic cleavage of VP0 into VP2 (28 kDa) and VP4 (8 kDa) occurs during encapsidation of the viral genome to produce the mature virus [1], [2]. During infection empty particles (hereafter termed natural empty particles) may also be produced, which resemble the mature virus in structure and antigenicity, but are inherently less stable [1]. The structural proteins are arranged in an icosahedral lattice of 12 pentameric building blocks which are the major structural intermediates for FMDV assembly and disassembly. The capsids are held together by electrostatic interactions, hydrogen bonds and weak hydrophobic interactions between the inter-pentameric subunits [3], [4] and unlike enterovirus capsids which release RNA by receptor-mediated uncoating [5], [6], FMDV capsids appear to release their genome by dissociation into pentamers at pH<7.0 and elevated temperatures. This instability translates into vaccines with limited shelf-life, necessitating a cold chain in many parts of the world where they are distributed.

Attempts to produce alternative vaccines have shown that intact virus particles stimulate the best immune response [7]. Picornavirus capsids can be synthesized using recombinant techniques by expressing (minimally) the P1 structural protein precursor and the 3C protease that cleaves it, since the capsid proteins spontaneously assemble to produce empty virus-like-particles(VLPs) [8]. The inherent problem is balancing the expression of P1 and 3C, the latter being toxic to cells, especially in the case of FMDV [2], [9], [10]. We demonstrate here the expression of FMDV VLPs at levels that are potentially viable commercially. We report the production of wild-type as well as stabilized VLPs, their characterization by X-ray crystallography and their ability to induce a sustained protective immunity in cattle.

## Results

### Expression strategies for VLPs and assessment of their stability

**Figure 1:**
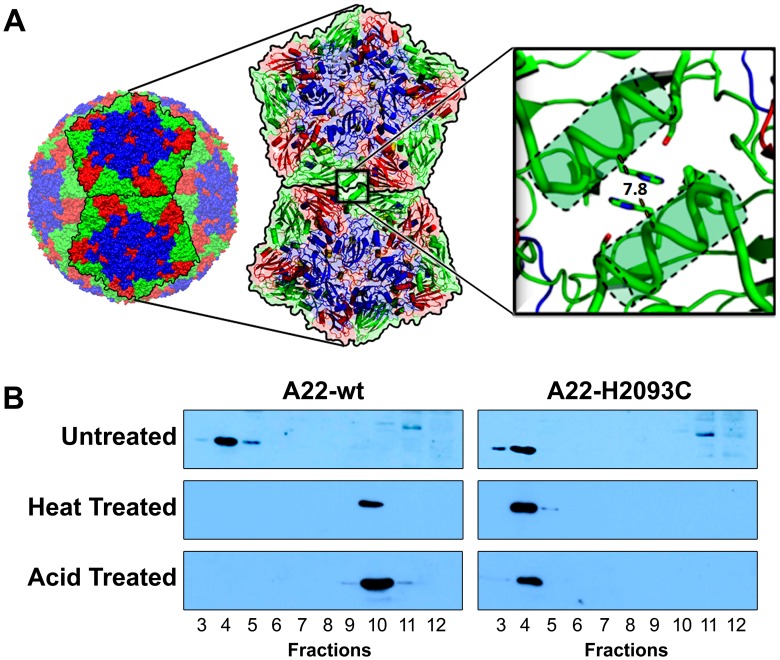
Rational design to produce stable empty capsids of FMDV A22. (a) The crystal structure of FMDV serotype A22 [2] was analysed as described in Materials and Methods. This led to the prediction and modelling of a potential disulphide bond between pentamers by mutation to cysteine of VP2 histidine residue 93, located on the α-helix at the two-fold symmetry axis. (b) Western blot analyses of A22-wt and A22-H2093C empty capsids, expressed from recombinant vaccinia viruses and sedimented through 15–45% sucrose gradients. Each gradient was fractionated from the bottom. FMDV capsid proteins were detected using an anti-FMDV strain A22 polyclonal antibody raised in guinea pigs. Crude extracts were left untreated, heated to 56°C for 2 h or acidified to pH 5.2 for 15 min. Following both heat and acid treatments A22-H2093C capsids remained intact and migrated to fraction 4, as before treatment, whereas A22-wt capsids fractured, remaining near the top of the gradient in fraction 10.

Three-dimensional structures for several serotypes of FMDV [2], [3], [11], [12] reveal the basis for the limited particle stability, highlighting specific atomic interactions along the interface between pentameric assemblies [4], [13]. This allowed us to pick histidine 93 of VP2, on the helix adjacent to the icosahedral 2-fold symmetry axis (**Figure 1a**) as a target for mutagenesis to a cysteine (H2093C) in order to form energetically favourable disulphide bonds across the inter-pentameric interface [14].

Initial production of empty capsids utilised infection of mammalian cells (RK13) with recombinant vaccinia viruses that encode a P1–3C expression cassette, where P1 (derived from FMDV A22) is either wild-type (vA22-wt) or carries the H2093C mutation in VP2 (vA22-H2093C). This cassette was flanked by 5′ and 3′ UTRs to permit translation from the FMDV IRES [15] (**Figure S1 in Text S1**). Expression of capped transcripts was driven by a T7 polymerase promoter [14]. Provision of the T7 polymerase by co-infection with vaccinia virus recombinant vTF7.3, effectively lowers the cytotoxicity due to 3C protease or its capsid product; use of an inducible promoter regulates the expression levels of the P1-2A-3C cassette compared to that of a constitutive promoter [9], [16]. Infected cells were pelleted, lysed and the extracts analysed using 15–45% sucrose gradient sedimentation. Gradient fractions were collected from the bottom of the tubes and analysed by western blot using anti-FMDV A22 serum. FMDV capsid proteins were detected in the bottom half of the gradients (fractions 4 in **Figure 1b** top panel), indicative of the ability of both the wild-type and mutant recombinant capsid proteins to assemble into empty particles [14].

To investigate the stability of the recombinant particles, aliquots (from fractions 4 of the wild-type and H2093C gradients shown in **Figure 1b** top panel) were subjected to either acidification or heating to 56°C for 2 h before repeat sedimentation on 15–45% sucrose gradients, with fractions analysed by western blot. Heat-treated A22-wt derived proteins remained near the top of the gradient in fraction 10 (**Figure 1b**, middle left panel) demonstrating dissociation. By contrast, proteins derived from heat-treated A22-H2093C were detected predominantly in fraction 4, showing that the mutant particles had withstood heating (**Figure 1b**, middle right panel). Acidification was to pH 5.2 with sodium acetate buffer. The samples were incubated for 15 min before sucrose gradient sedimentation. Analysis of gradient fractions by western blot showed that A22-wt capsids dissociated (**Figure 1b**, bottom left panel), whereas A22-H2093C capsids remained intact (**Figure 1b**, bottom right panel).

**Figure 2:**
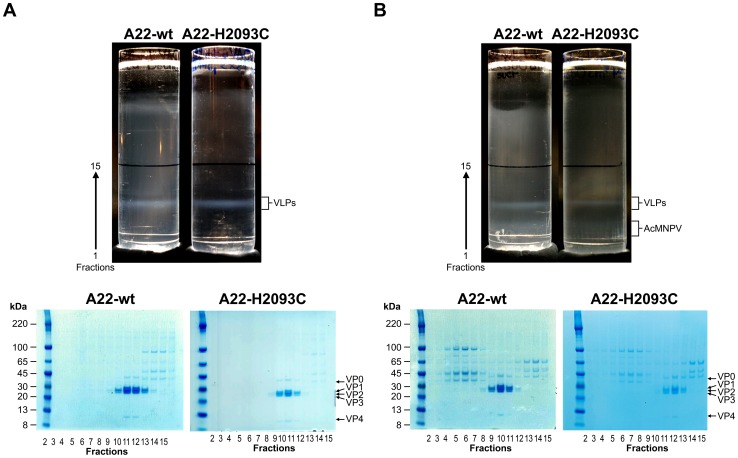
Sucrose gradient purification and SDS PAGE analysis of A22-wt and A22-H2093C recombinant capsids. The extracts from either 2×2125 cm^2^ roller flasks of HEK293T cells co-infected with recombinant vaccinia viruses, vA22-wt+vTF7-3 or vA22-H2093C+vTF7-3 (a) or from 300 ml culture of Sf9 cells infected with recombinant baculovirus virus, bA22-wt or bA22-H2093C (b) were each loaded onto a 36 ml 15–45% sucrose gradient and centrifuged at 21,000 rpm for 22 h at 12°C (SW32 rotor, Beckman). Bands were visualised by illumination of the gradients from the top with a halogen fibre optic light source (Schott). Fractionation of the lower halves of the gradients was in 13 aliquots of 1 ml (fractions 1–13) and 3 aliquots of 2 ml (fractions 14–16) collected from the bottom: 10 µl each from fractions 2 to 15 were analysed on 4–12% acrylamide Novex NuPAGE gels (Invitrogen).

To develop a practical method for novel vaccine production we explored the baculovirus expression system [17]. The same P1-3C cassettes were inserted into a baculovirus-compatible transfer vector, pOPINE [18]. In recombinant baculoviruses (bA22-wt and bA22-H2093C), capsid expression is driven by the baculovirus promoter p10. Capsid expression was optimised by reducing the activity and expression of 3C protease and thereby its toxic effects on the cell: this was achieved by site-directed mutagenesis in the vicinity of the 3C protease active site to reduce its activity and by insertion of a frameshift sequence upstream of the 3C gene to down-regulate its expression [19]. Following infection of suspension cultures of Sf9 cells [19], capsids were purified by a procedure similar to that used for mammalian cells. FMDV capsids produced in insect cells sedimented at the same position on 15–45% sucrose gradients as those produced using vaccinia virus in mammalian cells (**Figure 2a** top panel). A second lower band was observed on gradients loaded with the extract from insect cells (**Figure 2b** top panel). SDS-PAGE confirmed that the upper band harboured FMDV capsid proteins whilst the lower band contained proteins from baculovirus nucleocapsids (**Figure 2b** bottom panel). Yields ranged from 0.8 µg/ml for A22-H2093C to 1.2 µg/ml for A22-wt. VP0 was cleaved into VP2 and VP4 to a similar extent in recombinant A22 wt and H2093C. This type of cleavage had already been observed with A22 empty particles arising during an FMDV infection and progressed to completion on short term storage [2].

### Crystallographic analyses of VLPs

*In situ* room temperature X-ray crystallography [20] was used to determine the structure of both wild-type and mutant capsids produced using vaccinia virus at 2.2 Å and 2.9 Å resolution respectively. The crystals were essentially isomorphous to those obtained for both A22 virus and A22 natural empty particles [2], [21], and refinement, using strict 15-fold non-crystallographic symmetry and real space averaging gave reliable maps and models for both structures. Diffraction from crystals for baculovirus expressed particles was indistinguishable, demonstrating that the particles from both expression systems are iso-structural (data not shown).

The structures of recombinant A22 empty capsids were very similar to those previously reported for A22 virus and its natural empty particles [2], [21]. However, one region on the surface of the particle showed a significant structural difference: residues 171–181 of the VP3 GH loop adopt essentially identical folds in the two natural particles, whereas in wt and H2093C recombinant particles their structure is more extended (**Figure S2 in Text S1**) and almost identical to that seen in another serotype A virus, A10 [11]. We have previously shown that, for serotype O viruses, the VP3 GH loop conformation is modulated by changes in the adjacent VP1 GH loop [21], so it is possible that one or more amino acid sequence changes occurred in the highly variable disordered VP1 GH loop during native A22 virus replication and account for the repacking of the VP3 loop (no sequence changes were detected in the ordered portions of the native A22 virus capsid) [2].

**Figure 3:**
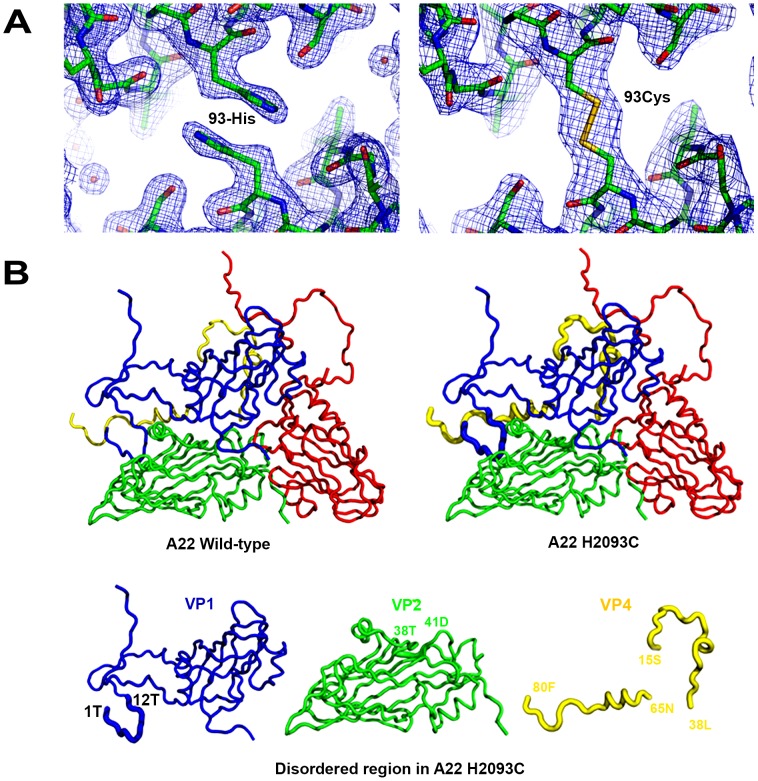
Structure analysis of A22-wt and A22-H2093C empty capsids. (a) The electron density map for A22-wt (left panel) presents the expected histidine side chains for VP2 residue 93 whereas the map for A22-H2093C (right panel) shows the disulphide density at the two-fold axis between pentamers. (b) Side by side ribbon drawings of the recombinant empty capsid protomers and beneath the individual VP1, 2 and 4 proteins. There was no difference between the A22-wt and A22-H2093C capsids on the exterior surface; significant disorder observed on the interior of the A22-H2093C capsids is shown as thickened lines and corresponds to the N terminus of VP1 (residues 1 to 12) and VP4 (residues 15 to 38 and 65 to 80) located near the 3-fold axes. To a lesser extent residues 38–41 in VP2 were also disordered.

The electron density map for the A22-H2093C mutant particles showed that the disulphide bond across the 2-fold axis relating two pentamers was correctly formed whilst A22-wt showed the expected histidine side-chain density (**Figure 3a**). A difference electron density map between the wt and H2093C recombinant particles (**Figure S3 in Text S1**) revealed, apart from this mutated residue, no significant features on the particle surface. Changes are however apparent on the interior of the particles, with VP4 being similarly ordered in the recombinant wt particle and the A22 virus structure [2] whereas only two residues of VP4 were visualized in the electron density map for H2093C (**Figure 3b**). It is possible that the greater rigidity of the H2093C particle inhibits movements required for VP4 to fully settle into the structure seen in mature virus following cleavage of VP0. Structural superimposition gives rms deviations in Cαs of the native and mutant recombinant particles of 0.36 Å (661 equivalent residues) and 0.4 Å (609 equivalent residues) respectively compared to the A22 virus.

### Immunogenicity of VLPs

**Figure 4:**
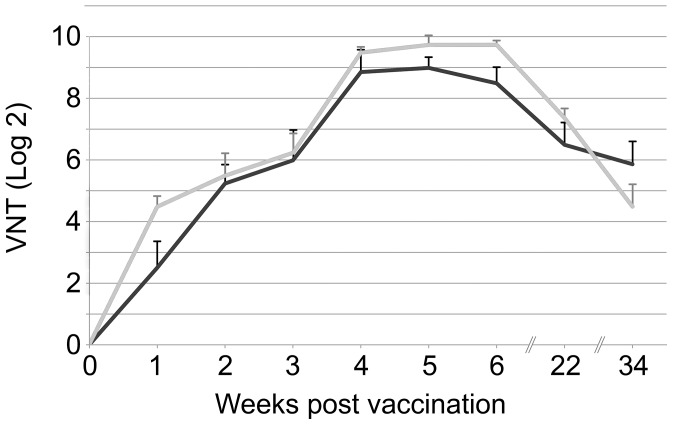
Virus neutralising antibody titres pre-challenge. The group mean virus neutralising antibody titres (Log2) for animals inoculated with A22-wt (black line) or A22-H2093C (grey line) are shown from week 0 to week 34. All animals were vaccinated on week 0 and week 3. Blood samples were taken at weekly intervals until week 6 and then on week 22 and finally on week 34 when challenge with live virus was carried out. Error bars represent the standard error of the mean.

To characterize the protective immunity induced by recombinant capsids produced with baculovirus, we immunised two groups of four out-bred cattle each with 12 µg of highly purified capsids formulated in oil adjuvant. One group received A22-wt and the other A22-H2093C capsids. All animals were re-immunised after 3 weeks. There was a rapid induction of neutralising antibodies after primary immunisation. The mean antibody titre of both groups was greater than 5.5 (Log_2_) which is considered protective [22]. There was a significant increase in virus neutralising antibody titres (VNT) post-boost and antibodies were maintained at high titres, greater than 6 (Log_2_), up to 22 weeks post-immunisation, but had reduced to pre-boost levels after 34 weeks in both groups of animals (**Figure 4**). Throughout the experiment there were no significant (P>0.05) differences in titres between animals vaccinated with wild-type empty capsids and those vaccinated with mutated empty capsids.

**Figure 5:**
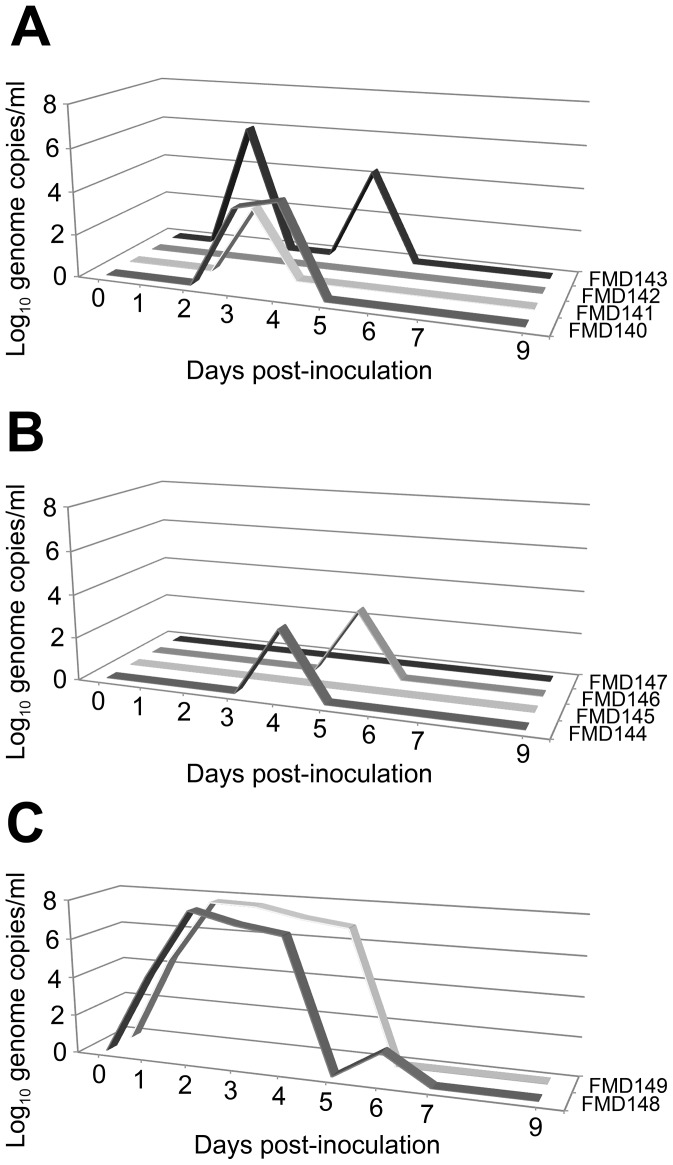
Virus genome detection. The quantity of viral genome (Log10/ml) was determined by quantitative real-time PCR in serum samples from each animal on days 0–7 and 9 post-challenge. Individual data for animals vaccinated with (a) A22-wt, (b) A22-H2093C and for non-vaccinated control animals (c). Clinical signs were detected in two of the four animals that had received wild-type empty capsid (FMD140 and FMD143) and in one of the four animals immunised with mutated capsids (FMD144). Both non-vaccinated control animals showed overt clinical signs.

At 34 weeks post immunisation the animals, plus two non-vaccinated control animals, were challenged by direct inoculation into the tongue with live A22 virus. Two of the four animals immunised with wild-type capsids and three of the four immunised with mutated capsids were fully protected using the criteria described in the European pharmacopeia [22]. The two control animals succumbed to full clinical signs (e.g. vesicular lesion of all four feet). Large quantities of viral genome were detected in their sera (greater than 6.5 log_10_ genomes/ml) for three or four days (**Figure 5c**). In contrast, lower quantities of viral genome were detected in the vaccinated animals (**Figure 5a and 5b**). The total amount of virus produced, estimated by computing the area under the curve (AUC) for copy number versus time, showed that there were no significant differences in AUC between animals vaccinated with A22-wt empty capsids and those vaccinated with A22-H2093C empty capsids (P = 0.23), whilst the AUC was significantly higher for non-vaccinated compared with vaccinated animals (P = 0.04).

## Discussion

Here we have demonstrated the production of safe, effective FMDV empty capsids that do not require bio-containment during manufacture. Furthermore, enhanced stability of the empty capsids will reduce losses during production, storage and transport whilst maintaining antigenic structure and immunogenicity. In addition the complete absence of FMDV non-structural proteins from the vaccine formulation will allow the development of diagnostic tests to discriminate between infected and vaccinated animals (DIVA).

Disulphide bonds are used to stabilise many extracellular proteins and also certain virus capsids [23]. Such covalent cross-links are more robust than the non-covalent interactions that generally hold protein assemblies together. Here we have rationally engineered a disulfide bond by mutating a single histidine residue at position 93 of VP2 located at the icosahedral 2-fold axis between adjacent pentamers [4]. Baculovirus expressed wild-type and stabilised capsids produced equivalent titres of neutralising antibodies, following a standard immunisation regimen, over a 34 week period post immunisation. These results inform the debate on the effect of increased antigen stability on immunogenicity. Delamarre et al [24] showed that for two proteins with the same T cell and B cell epitopes but with different susceptibilities to lysosomal proteolysis, mediated by single point mutations, less digestible forms induced more efficient T cell priming and antibody responses. In contrast, recent studies with a model antigen in mice suggested that enhanced conformational stability resulted in reduced antigenicity [25]. Although as yet we do not know if forming a disulphide bridge will be possible for all serotypes, especially in the baculovirus expression system, our results demonstrate that capsid stability can be augmented without compromising immunogenicity and this might be a general tactic for improving vaccine efficacy. The rational structure-based approach initiated here should in principle allow the tuning of these parameters to match the particular circumstances of different viruses. For instance, enhancing the stability of capsids for highly prevalent FMDV serotype O, which are more labile than those of A22 [26]. Recent work on EV71 has demonstrated that maintaining the proper positions of the 2-fold helices (which harbour the H2093C mutation in FMDV) is essential for maintaining native antigenicity [6], suggesting that the approach we have demonstrated here may be applicable across a wide range of human and animal picornaviruses, including polioviruses and coxsackieviruses.

## Materials and Methods

### Vaccinia virus transfer vectors

An expression cassette based on the sequence of FMDV A22 Iraq was designed (**Figure S1 in Text S1**), synthesized de novo (Geneart) and cloned into the vaccinia virus transfer vector pBG200 [9] downstream of the T7 promoter. Substitution of a BstEII-SpeI fragment with a sequence encoding the H2093C mutation converted the pBG200-A22-wt plasmid to pBG200-A22-H2093C.

### Generation and selection of vaccinia virus recombinants

The recombinant viruses were made by transfecting pBG200-A22-wt and pBG200-A22–H2093C into CV-1 cells infected with vaccinia virus (VV) strain WR. Recombinant VVs (with an interrupted thymidine kinase gene) were selected in HuTK-143 cells using 5-bromo-2-deoxyuridine. Three rounds of plaque purification in conjunction with screening by PCR using FMDV-specific primers were carried out to get stable recombinant VVs. These were amplified in RK13 cells and virus stocks titrated by plaque assay on BS-C-1 cells.

All mammalian cells were grown in DMEM supplemented with 10% FCS and appropriate antibiotics at 37°C.

### Initial sedimentation of empty capsids produced via vaccinia virus

A single 175 cm^2^ flask of RK13 cells was dually infected with either vA22-wt or vA22-H2093C at an MOI 10 and vTF7.3 at an MOI 5. After 24 h cells were harvested by centrifugation at 2,000 g for 5 min at 4°C and the pellet resuspended in 1 ml 0.5% Ipegal (Sigma) in 40 mM sodium phosphate, 100 mM NaCl pH 7.6. The sample was incubated on ice for 20 min, clarified, loaded onto a 15–45% sucrose gradient and spun for 20 h at 22,000 rpm (SW41 rotor, Beckman) at 12°C. Each gradient was fractionated into 12 fractions of 1 ml and aliquots were analysed by western blotting.

### Thermo-stability/pH stability assay

A 200 µl aliquot of the empty capsid-containing fraction identified during the initial sedimentation experiment (see above), was diluted 1/3 either (i) with phosphate buffer pH 7.6 and incubated in a water bath at 56°C for 2 h or (ii) with 50 mM sodium acetate buffer pH 4.6, to give a final pH of 5.2 and incubated at room temperature for 15 min before neutralisation with NaOH. Treated samples were loaded onto 15–45% sucrose gradients, centrifuged and fractionated as above. Each fraction was precipitated with an equal volume of saturated ammonium sulphate overnight at 4°C. Precipitates were collected by centrifugation at 16,000 g for 15 min at 4°C and analysed by western blot.

### Expression and purification of empty capsids from mammalian cells

HEK293 cells grown in 2×2125 cm^2^ roller flasks were dually infected as described above. After 20 h, cells were pelleted at 3,500 g for 30 min at 4°C. Pellets were resuspended in phosphate buffer and lysed with 0.5% Igepal on ice for 20 min. Lysates were clarified at 10,000 g for 20 min at 4°C and the resulting pellets resuspended in a small volume of buffer for re-extraction with 1 volume of chloroform. The aqueous phases were pooled with the clarified extracts and pelleted over 30% sucrose cushions at 105,000 g for 5 h at 12°C. Pellets were resuspended in a small volume of buffer, treated with 200 mg/ml RNAse A in the presence of 0.1% Igepal for 30 min on ice, clarified and loaded onto a 15–45% sucrose gradient. Following centrifugation at 54,000 g for 22 h at 12°C, the gradient was fractionated and fractions analysed by SDS-PAGE. Sucrose was removed by desalting with a spin column (Zeba, Pierce) and samples concentrated by ultrafiltration (Amicon).

### Baculovirus transfer vector

pTri-EX-derived plasmid pOPINE was used for In-Fusion cloning [18] of the FMDV coding sequence from pBG200-A22-wt resulting in pOPINE-A22-wt [19]. An overlapping PCR procedure which exchanged a P1 region for a fragment bearing the H2093C mutation resulted in plasmid pOPINE-A22-H2093C. Subsequent alterations within the P1-2A-3C expression cassette in order to down-regulate the 3C protease were as described [19].

### Generation of baculovirus recombinants and insect cell expression

Sf9 cells were grown in Insect-XPRESS (Lonza) supplemented with 2% FCS and antibiotics at 27.5°C. Transfer vector and AcMNPV bacmid KO1629 (0.5 µg of each) were mixed in the presence of 3 µl Fugene (Roche) for 20 min at room temperature and used to transfect Sf9 cells at a density of 1.2×10^6^/well in a 6-well plate. Since baculovirus DNA with gene knockout 1629 will not initiate an infection unless rescued by recombination with a baculovirus transfer vector, the AcMNPV harvested in the culture supernatant after 5 days was 100% recombinant virus [19]. Virus stocks were produced by infecting Sf9 cells at a confluence of 70% with 200 µl recombinant per 175 cm^2^ flask and harvested from culture supernatants after 5 days. For the expression of empty capsids, Sf9 cells at a density of 1–2 10^6^/ml were infected with 1/10 volume of baculovirus stock. After 3 days virus extraction was as described for mammalian cells except that lysis was with 1% Triton X-100 in the presence of 5 µl/ml protease inhibitor cocktail (Sigma).

### Molecular modelling and disulphide design

The crystal structures of FMDV serotypes A10 [11], A22 [2] and O1bfs [3] were inspected and amino acid residues involved in inter-pentameric interactions were identified. An energetically favourable disulphide bond was predicted by manually measuring the pair-wise Cα_i_-Cα_j_ and corresponding Cβ_i_-Cβ_j_ distances using COOT [27].

### Crystallisation and data collection

Crystals were grown by the sitting-drop vapour diffusion method in Crystalquick X plates (Greiner Bio-One) using 100 nl virus plus 100 nl precipitant dispensed with a Cartesian liquid dispensing robot as described previously [28]. Micro-crystals of A22-wt empty capsids (3 mg/ml) with average dimensions of 50×50×5 µm^3^ and A22-H2093C (2.3 mg/ml) with average dimensions of 30×30×5 µm^3^ grew within 1 week at 294K with 4 M ammonium acetate, 100 mM bis-Tris Propane, pH 7.0. Optimisation by varying the concentration of precipitant and pH around the initial condition produced sufficient crystals for structural solution. A 20×20 µm^2^ beam (λ = 0.9778 Å; I24 micro-focus beamline, Diamond), was used for *in situ* diffraction image collection [20] at 294 K on a Pilatus 6 M detector.

### Structure determination, refinement and phasing

The structures of A22-wt and A22-H2093C were solved by molecular replacement. The orientation of the particles (obtained from a self-rotation function) was found to be the same as for the native A22 virus structure (PDB id: 4GH4). Hence the coordinates and non-crystallographic symmetry (NCS) operators from native virus were used for the refinement. Initial estimates of phases were obtained by rigid body refinement with CNS [29]. Iterative positional and B-factor refinement (via CNS) used strict NCS constraints (**Table S1 in Text S1**). Phases were further improved by 15-fold cyclic NCS averaging using the General Averaging Program (GAP, D Stuart and J. Grimes, unpublished). There was good agreement between the observed data and those calculated from the averaged electron density map of R = 10.4% and CC = 97% for the wild-type and R = 12.3% and CC = 95% for the mutant. Model building used COOT [27].

### Cattle vaccination, challenge and clinical assessment

Two groups of four 100 to 150 kg Holstein Friesian calves were vaccinated with either A22-wt or A22-H2093C capsids. Each animal received 12 µg of purified capsid formulated in oil adjuvant (Seppic 206B) as an intramuscular injection on week 0 and week 3 of the study. All eight animals plus two non-vaccinated control animals were needle challenged intradermolingually with 1×10^5^ TCID_50_ of cattle adapted FMDV A22 on week 34. Animals were examined clinically and blood sampled from the day of challenge until day 9. Cattle were considered protected if lesions could not be detected at sites distal from the inoculation point. Animal experimentation was approved by the Pirbright Institute (PI) ethical review board under the authority of a Home Office project licence in accordance to the Home Office Guidance on the Operation of the Animals (Scientific Procedures) Act 1986 and associated guidelines.

### Titration of neutralising antibodies

Sera from the 8 immunised cattle and control sera from 2 non-vaccinated animals were prepared from blood samples. Their neutralising activities were determined as reported previously; testing was in duplicate, in serial 2-fold dilutions and endpoints calculation was made as described [22]. Titres of FMDV-specific antibodies are expressed as the reciprocal value of the highest dilution giving ≥50% neutralisation of homologous virus growth.

### qRT-PCR analysis of viral load in sera

Total nucleic acid was extracted from 200 µl of serum and automated reverse transcription procedures were performed incorporating homologous FMDV RNA standards. Real-time PCR amplification was performed using A22 specific primers and standard curves constructed to provide a measure of the number of FMDV genome copies [30].

### Statistical analysis

Virus neutralisation titres were analysed using linear mixed models including time since vaccination and vaccine type (wt or H2093C empty capsids) as factors and animal as a random effect. Model selection proceeded by stepwise deletion of non-significant (P>0.05) terms, starting from a model including time since vaccination and vaccine and an interaction between them. The total amount of virus produced following challenge was estimated for each animal by computing the area under the curve (AUC) for copy number versus time using the trapezium rule. A Wilcoxon rank-sum test was used to check for significant (P<0.05) differences in AUCs, first between animals vaccinated with A22-wt or A22-H2093C empty capsids and second between vaccinated (with either capsid) and non-vaccinated animals.

## Supporting Information

Text S1 **Supporting Information.** In Text S1, Figure S1 shows the configuration of the FMDV expression cassette cloned into vaccinia virus transfer vector pBG200. Figure S2 shows the extended structure of the VP3 GH-loop and its sequence alignment to another A serotype strain. Figure S3 shows disordered regions in the recombinant A22-H2093C capsid. Table S1 provides X-ray data collection and refinement statistics.
(DOCX)
