# Supplementary material for: Rational Engineering of Recombinant Picornavirus Capsids to Produce Safe, Protective Vaccine Antigen
Source: PLoS Pathog. 2013 Mar 27;9(3):e1003255. doi: 10.1371/journal.ppat.1003255 (PMC3609824; doi:10.1371/journal.ppat.1003255)
Supplement: Text S1 — Supporting Information. In Text S1, Figure S1 shows the configuration of the FMDV expression cassette cloned into vaccinia virus transfer vector pBG200. Figure S2 shows the extended structure of the VP3 GH-loop and its sequence alignment to another A serotype strain. Figure S3 shows disordered regions in the recombinant A22-H2093C capsid. Table S1 provides X-ray data collection and refinement statistics. (DOCX) [file ppat.1003255.s001.docx]

**Supporting Information**

**
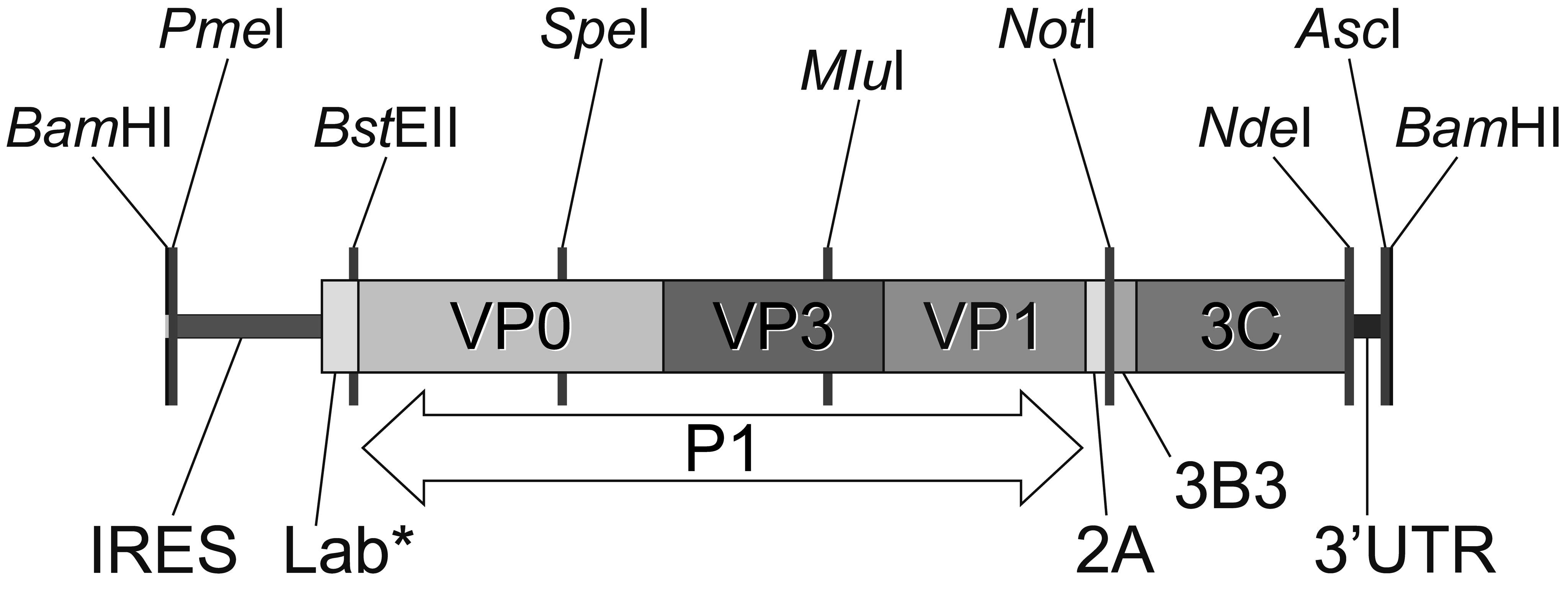
**

**Figure S1. Configuration of the FMDV expression cassette cloned into vaccinia virus transfer vector pBG200.** The coding sequence is comprised of P1, the precursor of the capsid proteins, the 2A sequence which mediates a co-translational cleavage and the 3C protease with the adjacent 3B3 gene. Upstream of P1 is a part of the FMDV 5’UTR region which spans nucleotides 640-1180 and comprises the internal ribosome entry site (IRES) and the sequence of the Lab leader protease with a mutated initiation codon (Lab*). Most IRES-mediated initiation of translation occurs at the next initiation coding -for deleted gene Lb- [[1](#_ENREF_1)], presently placed at the start of the P1 sequence. Downstream of 3C is the FMDV 3’UTR followed by a 20 nucleotide long polyA tail, which both participate in enhanced translation from the FMDV IRES. Silent mutations were used for the introduction of internal restrictions sites allowing the substitution of various components of the expression cassette and facilitating mutagenesis of P1.


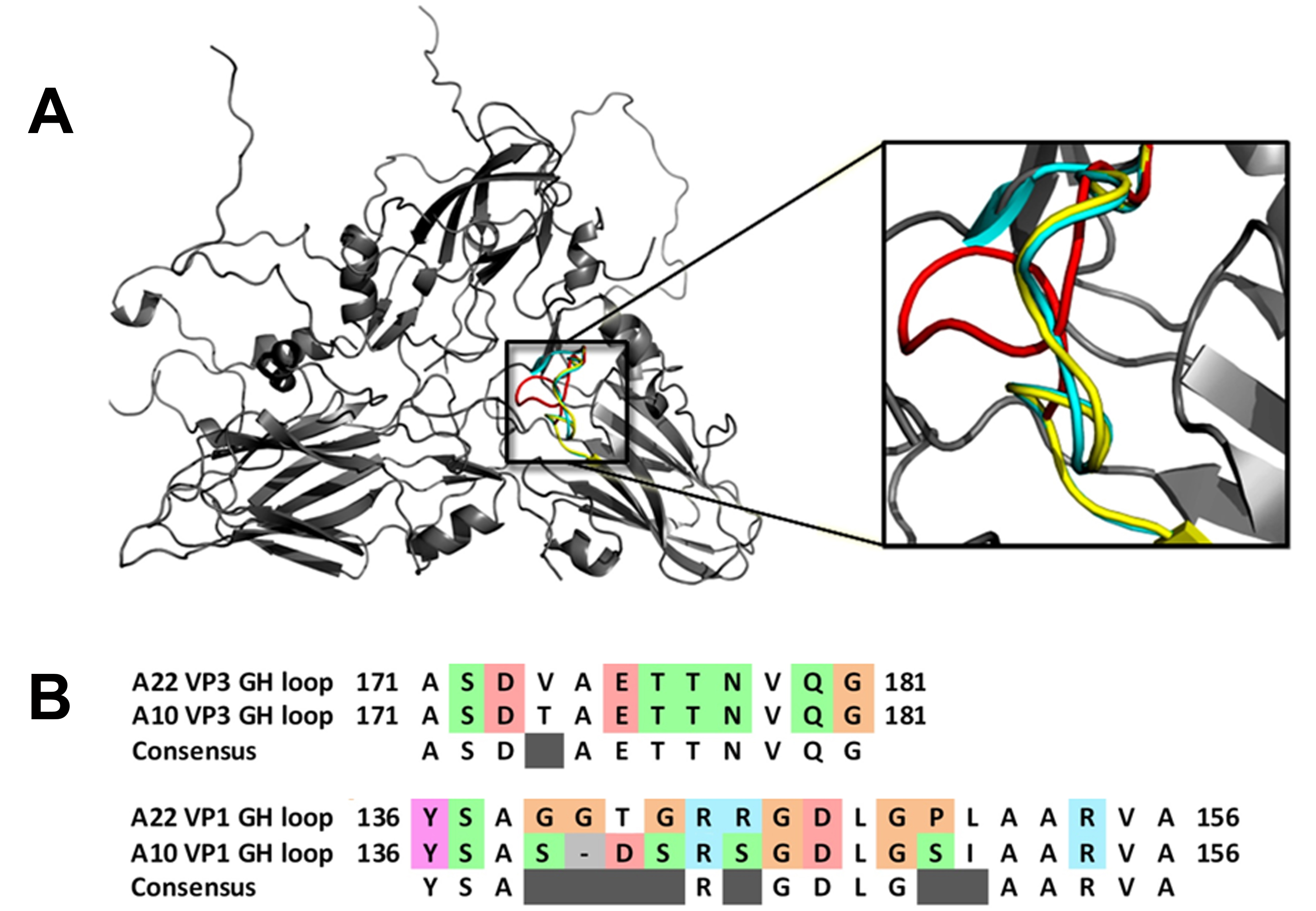


**Figure S2.** **The extended structure of the VP3 GH-loop and its sequence alignment to another A serotype strain.** (a) The recombinant wt and mutant structures were for the most part similar to the A22 native virus; however the VP3 GH loop (residues 174-182) in recombinant particles (yellow) is more extended compared to that of the native virus (red). This has been observed previously in another serotype-A virus, A10 [[2](#_ENREF_2)] (cyan). (b) Sequence alignment of theVP1 and VP3 GH loops between the A22 and A10 serotypes. The VP3 GH loop conformation is modulated by changes in the adjacent VP1 GH loop [[3](#_ENREF_3)], so it is possible that one or more amino acid sequence changes in the highly variable disordered VP1 GH loop of the native A22 structures account for the repacking of the VP3 loop.

**
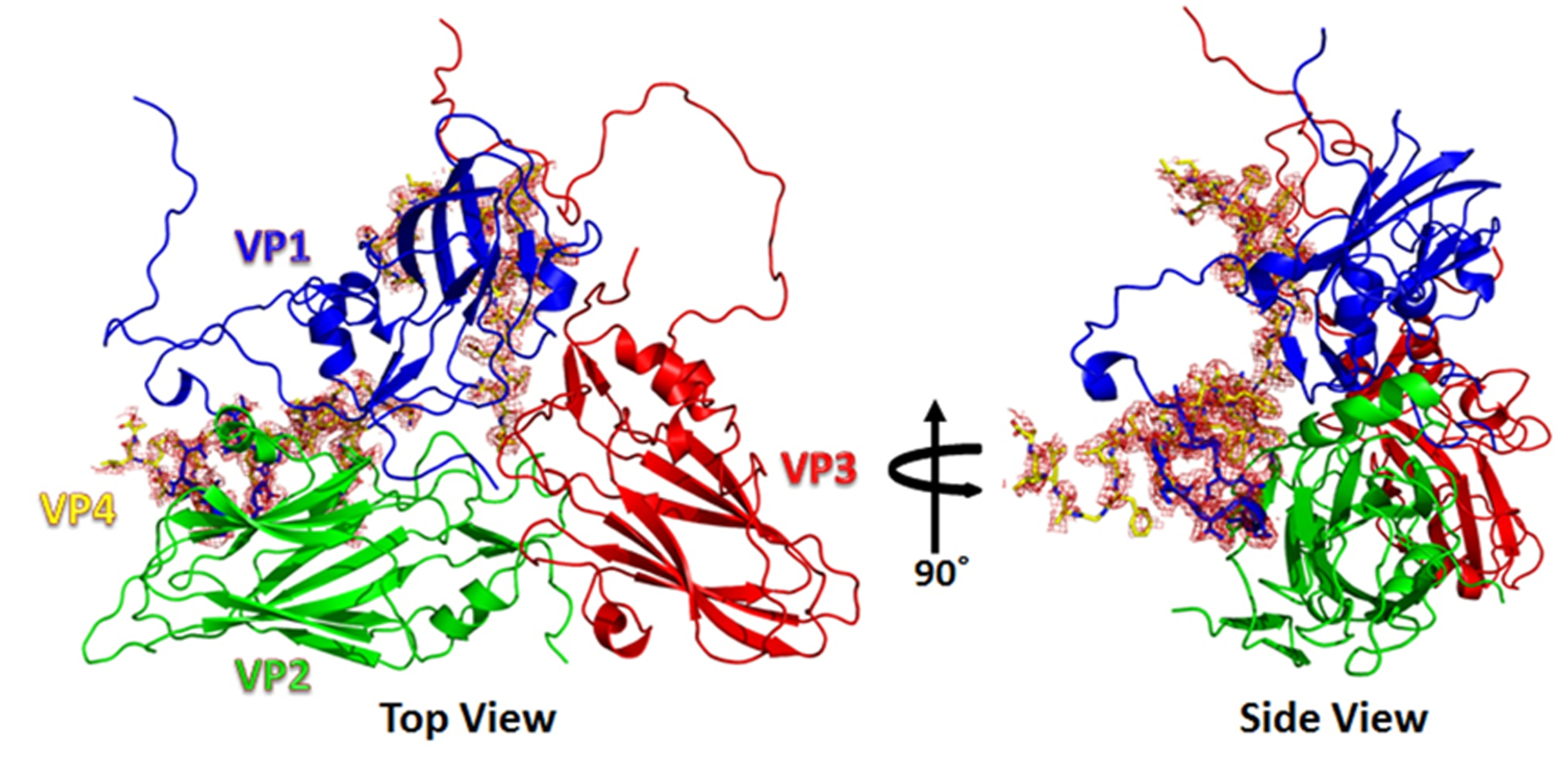
**

**Figure S3.** **Disordered regions in the recombinant A22-H2093C capsid.** The structures of the recombinant A22-wt and A22-H2093C mutant were similar to that of the A22 virus structure on the exterior surface. However on the interior surface, the entire VP4 was found to be disordered. This was verified by an electron density difference map calculated from the amplitudes of recombinant wt and mutant empty capsids Fo_mut_-Fo_wt_. The disordered regions are shown in red density. The N-terminus of VP1 (residues 1 to 12) and residues 38-41 in VP2 were also disordered. Nevertheless the disordered regions are in the interior of the capsid and do not affect the antigenic properties of the mutant virus.

**Table S1: X-ray data collection and refinement statistics**

| **X-ray source** | **Diamond, beamline I24** | |
| --- | --- | --- |
| **Data set** | **A22 wild type** | **A22 VP2 H93C** |
| Wavelength ( Å ) | 0.9778 | 0.9778 |
| Number of crystals/positions | 47(125) | 77 (191) |
| Number of images | 991 | 764 |
| Rotation per image (˚) | 0.1 | 0.1 |
| Space group | *I222* | *I222* |
| Unit cell (Å) | *a* = 327.6, *b* = 341.4,  *c* = 363.7 | *a* = 328.0, *b* = 341.5,  *c* = 363.4 |
| Resolution range (Å)^a^ | 50 – 2.1 (2.18-2.10) | 50 – 2.9 (3.0-2.9) |
| Unique reflections | 869280 (29881) | 353968(23484) |
| Completeness (%) | 74.6 (25.8) | 79.7 (53.2) |
| Multiplicity | 2.7 (1.2) | 2.2 (1.6) |
| Average I/σI | 3.5 (0.5) | 2.2 (0.7) |
| R_merge_ ^b^ | 0.244 | 0.407 |
| Refinement statistics: | | |
| Resolution range (Å) | 50 – 2.1 | 50 – 2.9 |
| No. of reflections(working/test) | 814431/43049 | 334790/17689 |
| R-factor(R_work_/R_free_)^c^ | 0.190/0.193 | 0.235/0.239 |
| Protein atoms | 5199 | 4793 |
| Non protein atoms | 349 | 58 |
| r.m.s.d. bond length (Å) | 0.014 | 0.006 |
| r.m.s.d. bond angle (°) | 1.8 | 1.3 |
| Average B-factor (protein/water Å^2^]) | 31/42 | 35/39 |

^a^Numbers in brackets for outer resolution shell

^b^Rmerge = ∑_hkl_∑_j_|I_hkl, merged_| - |I_hkl, j_|| / ∑_hkl_N|I_hkl, merged_|, where j=1, ... , N for N datasets.^c^R_work_ and R_free_ are defined by R=_hkl_*F_obs_|−* *F_calc_*  _hkl_* *|F_obs_|, where *h,k,l* are the indices of the reflections (used in refinement for R_work_; 5%, not used in refinement, for R_free_), F_obs_ and F_calc_ are the structure factors, deduced from measured intensities and calculated from the model, respectively.

**References cited in Supporting Information**

1. Belsham GJ (1992) Dual initiation sites of protein synthesis on foot-and-mouth disease virus RNA are selected following internal entry and scanning of ribosomes in vivo. EMBO J 11: 1105-1110.

2. Fry EE, Newman JW, Curry S, Najjam S, Jackson T, et al. (2005) Structure of Foot-and-mouth disease virus serotype A10 61 alone and complexed with oligosaccharide receptor: receptor conservation in the face of antigenic variation. J Gen Virol 86: 1909-1920.

3. Curry S, Fry E, Blakemore W, Abu-Ghazaleh R, Jackson T, et al. (1996) Perturbations in the surface structure of A22 Iraq foot-and-mouth disease virus accompanying coupled changes in host cell specificity and antigenicity. Structure 4: 135-145.
